# miR‐15a‐5p Regulates Ferroptosis in Glioblastoma by Targeting GLS2

**DOI:** 10.1002/cns.71097

**Published:** 2026-08-30

**Authors:** Yanyan Yu, Helan Yuan, Zikun Fang, Jinyuan Liao, Yancong Yang, Jun Liu, Wenjin Wei, Qiuhua Jiang

**Affiliations:** ^1^ Department of Neurosurgery, The Affiliated Ganzhou Hospital, Jiangxi Medical College Nanchang University Ganzhou Jiangxi China; ^2^ JXHC Key Laboratory of Rare Earth and Glioma Prevention and Treatment Ganzhou China; ^3^ Department of Neurosurgery The Second Affiliated Hospital of Nanchang University Nanchang Jiangxi China; ^4^ Ganzhou Key Laboratory of Molecular Medicine The Affiliated Ganzhou Hospital of Nanchang University Ganzhou Jiangxi China

**Keywords:** ferroptosis, glioblastoma, GLS2, miR‐15a‐5p

## Abstract

**Aims:**

Glioblastoma (GBM) remains the most lethal primary brain tumor, and the molecular mechanisms driving its aggressiveness are incompletely understood. Recent multi‐omics analyses have identified miR‐15a‐5p as one of the core microRNAs most closely associated with GBM. This study aimed to investigate the functional role and mechanism of miR‐15a‐5p in GBM pathogenesis.

**Methods:**

Expression of miR‐15a‐5p was analyzed in GBM tissues and cell lines. Genetic silencing experiments were performed to assess cell proliferation, migration, and tumor growth across multiple GBM cell lines and in an intracranial xenograft model. Ferroptosis‐related changes were evaluated, and target validation was conducted using luciferase reporter assays. Rescue experiments were performed by co‐modulating miR‐15a‐5p and GLS2.

**Results:**

miR‐15a‐5p was significantly overexpressed in GBM tissues and cell lines, and high expression correlated with poor patient survival. Genetic silencing of miR‐15a‐5p suppressed GBM cell proliferation and migration and inhibited tumor growth in vivo. Mechanistically, miR‐15a‐5p directly targeted the 3′UTR of glutaminase 2 (GLS2). Knockdown of miR‐15a‐5p induced ferroptosis‐related features, including GPX4 downregulation, ACSL4 upregulation, iron accumulation, lipid peroxidation, and mitochondrial contraction. These effects were reversed by GLS2 knockdown in both in vitro and in vivo rescue experiments.

**Conclusion:**

The miR‐15a‐5p/GLS2 axis represents a previously unrecognized mechanism by which GBM cells resist ferroptosis. These findings not only define a novel mechanism underlying ferroptosis regulation in GBM but also identify the miR‐15a‐5p/GLS2 axis as a potential entry point for ferroptosis‐based therapeutic intervention. Targeting this axis may provide a rational strategy to modulate ferroptosis and suppress GBM progression.

## Graphical abstract

Schematic diagram of the mechanism by which miR‐15a‐5p regulates glioblastoma progression through targeting GLS2 and modulating ferroptosis. miR‐15a‐5p regulates the ferroptosis pathway by targeting and inhibiting glutaminase 2 (GLS2) expression: Downregulation of miR‐15a‐5p leads to upregulation of GLS2, which promotes lipid peroxidation, accompanied by elevated levels of ferrous ions (Fe^2+^), malondialdehyde (MDA), and acyl‐CoA synthetase long‐chain family member 4 (ACSL4), as well as decreased expression of glutathione peroxidase 4 (GPX4), ultimately inducing ferroptosis in glioblastoma (GBM) cells. Activation of ferroptosis further suppresses the proliferation and migration of GBM cells, reduces tumor volume in tumor‐bearing mice, and thereby blocks the progression of glioblastoma.

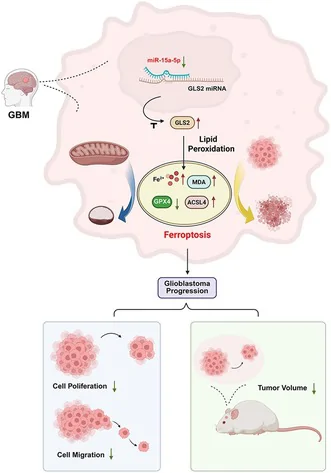

## 1 Introduction

Glioblastoma (GBM) is the most common and aggressive primary malignant brain tumor in adults, with a median overall survival of approximately 15 months even after maximal surgical resection, radiotherapy, and temozolomide chemotherapy [1, 2, 3]. The relentless progression of GBM reflects its extraordinary biological complexity, including pronounced genetic heterogeneity, infiltrative growth patterns, and intrinsic resistance to conventional therapies [4, 5, 6]. Elucidating the molecular mechanisms that drive GBM pathogenesis is therefore essential for developing more effective treatment strategies.

Ferroptosis, a form of regulated cell death characterized by iron‐dependent lipid peroxidation, has emerged as a promising therapeutic vulnerability in GBM [7, 8, 9]. In contrast to apoptosis, which is frequently disabled and confers broad therapeutic resistance in GBM, targeting ferroptosis offers a particularly appealing strategy by bypassing apoptotic blockade, exploiting GBM's intrinsic metabolic vulnerabilities, and potentiating anti‐tumor immunity [10, 11, 12]. Consistent with this concept, preclinical studies have shown that pharmacological stimulation of ferroptosis inhibits GBM growth in vitro and in vivo [13].

However, GBM cells can evade ferroptosis through multiple mechanisms, including upregulation of GPX4 and SLC7A11, as well as dysregulation of iron and lipid metabolism [14, 15]. Nevertheless, the upstream regulatory mechanisms that govern ferroptosis escape in GBM remain incompletely understood, and identifying context‐dependent regulators of this pathway may provide new therapeutic insights. Among these regulatory mechanisms, post‐transcriptional control mediated by microRNAs (miRNAs) has increasingly been recognized as an important layer of ferroptosis regulation [16]. Recent multi‐omics analyses identified miR‐15a‐5p as one of the five core miRNAs most closely associated with GBM pathogenesis [17] suggesting that it may play an important role in GBM biology. Moreover, evidence from other biological contexts has implicated miR‐15a‐5p in the regulation of oxidative stress and iron metabolism, further supporting its potential relevance to ferroptosis [18, 19]. However, current evidence regarding miR‐15a‐5p in GBM remains very limited. To date, only one in vitro study using a single cell line has reported that miR‐15a‐5p promotes proliferation and invasion in T98G cells by targeting CADM1 [20]. Collectively, these observations suggest that miR‐15a‐5p may represent a context‐dependent regulator linking GBM progression to ferroptosis escape. Whether miR‐15a‐5p is clinically relevant in GBM, whether it contributes to tumor progression in vivo, and most importantly, whether it regulates ferroptosis in GBM and through what molecular mechanism, all remain unknown.

Therefore, miR‐15a‐5p represents a compelling candidate regulator of ferroptosis in GBM. Based on these observations, we hypothesized that miR‐15a‐5p may act as a post‐transcriptional regulator of ferroptosis in GBM. To test this hypothesis, we integrated clinical analysis, in vitro functional assays, and in vivo validation to investigate the role of miR‐15a‐5p in GBM. Here, we demonstrate that miR‐15a‐5p directly targets glutaminase 2 (GLS2), thereby suppressing ferroptosis and promoting GBM progression. These findings identify the miR‐15a‐5p/GLS2 axis as a novel regulatory mechanism of ferroptosis in GBM and highlight a potential therapeutic vulnerability for ferroptosis‐based intervention.

## 2 Materials and Methods

### 2.1 Study Design and Ethics

This study was approved by the Ethics Committee of Ganzhou People's Hospital (human samples: PJB2025‐376‐01; animal studies: PID2025‐005‐01). All human participants provided written informed consent. Animal experiments complied with national laboratory animal guidelines (SYXK (Gan) 2023‐0006). The maximal tumor burden permitted was 1.5 cm in maximum diameter; no animals exceeded this limit.

### 2.2 Clinical Specimens Collection

Tumor tissues were collected from 20 patients (10 low‐grade glioma, 10 GBM) undergoing surgical resection in Ganzhou People's Hospital. Six nontumor brain tissues served as controls. All participants signed the informed consent form. The study was in conformity with the Declaration of Helsinki and approved by Ganzhou People's Hospital's Ethics Committee (Approval No. PJB2025‐376‐01).

### 2.3 Bioinformatic Analysis

Expression data for miR‐15a and corresponding survival information were obtained from the Chinese Glioma Genome Atlas (CGGA) database (http://www.cgga.org.cn). Analysis was performed using the miRNA dataset (microRNA‐array_198). Patients were divided into high‐ and low‐expression groups based on the median level of miR‐15a expression. Overall survival was analyzed using the Kaplan–Meier method with the log‐rank test. Potential target genes of miR‐15a‐5p were predicted using TargetScanHuman version 8.0 (http://www.targetscan.org/).

### 2.4 Cell Culture and Transfection

Human GBM cell lines (LN229, U87MG, U251, and T98G) and a human astrocyte line (HA) were cultured in DMEM (Gibco, Carlsbad, CA, USA) with 10% fetal bovine serum. For in vitro functional assays, LN229 and U87MG cells were transfected with miR‐15a‐5p inhibitor or negative control using TSanofectV2 transfection reagent (Tsingke, Beijing, China). For in vivo experiments, LN229‐Luc cells were transduced with lentiviral particles carrying miR‐15a‐5p‐shRNA or GLS2‐shRNA (GeneChem, Shanghai, China) to generate stable knockdown lines. Knockdown efficiency was confirmed by RT‐qPCR and Western blot. Target sequences are listed in Table S1. Ferrostatin‐1 (Fer‐1; MedChemExpress, Monmouth Junction, NJ, USA) was used at a final concentration of 2 μM for ferroptosis rescue experiments.

### 2.5 RNA Analysis

Total RNA was extracted using a commercial kit (Vazyme, Nanjing, China). RT‐qPCR was performed on a 7300 Real‐Time PCR System (Thermo Fisher Scientific, Waltham, MA, USA). Expression levels were calculated using the 2^−ΔΔCt^ method with U6 as internal control. Primer sequences are listed in Table S1.

### 2.6 Western Blotting

Total protein was extracted with RIPA lysis buffer (Beyotime, Shanghai, China) and quantified using a BCA kit (Beyotime). Protein samples were separated by SDS‐PAGE and transferred onto nitrocellulose membranes (Cytiva, Marlborough, MA, USA). Membranes were blocked with blocking buffer (Beyotime) at room temperature (RT) for 30 min and then incubated with primary antibodies at 4°C overnight. After washing with TBST, membranes were incubated with HRP‐conjugated secondary antibodies at RT for 1 h. Signals were visualized using an enhanced chemiluminescence (ECL) kit (Servicebio, Wuhan, China) and imaged using a Bio‐Rad imaging system. Band intensities were quantified using ImageJ (version 1.53k, NIH, Bethesda, MD, USA). Detailed antibody information is listed in Table S1.

### 2.7 Immunofluorescence

Cells were seeded onto coverslips in 24‐well plates. After 24 h of culture, cells were fixed with methanol at −20°C for 10 min and blocked with 6% BSA in PBS at RT for 30 min. Samples were incubated with primary antibodies at 4°C overnight, followed by incubation with fluorochrome‐conjugated secondary antibodies (Panovue, Beijing, China) at RT for 60 min in the dark. Nuclei were counterstained with DAPI (Servicebio). Fluorescence images were acquired using a confocal microscope (ZEISS LSM 900, Jena, Germany), and average fluorescence intensity was quantified using ImageJ. Detailed antibody information is listed in Table S1.

### 2.8 Dual‐Luciferase Reporter Assay

HEK293T cells were co‐transfected with wild‐type or mutant GLS2 3′UTR reporter constructs and miR‐15a‐5p mimics or negative control. Luciferase activity was measured using a dual‐luciferase reporter assay kit (Hanheng Biotechnology, Shanghai, China).

### 2.9 Cell Proliferation Assays

For CCK‐8 assays, cells were seeded in 96‐well plates at a density of 2 × 10^3^ cells per well. At specified time points, 10 μL of CCK‐8 reagent (Servicebio) was added and incubated for 2 h. Absorbance at 450 nm was measured using a microplate reader. For EdU assays, cells were seeded onto coverslips in 24‐well plates. After 24 h, EdU working solution (Beyotime) was added for 2 h. Cells were then fixed, permeabilized, and stained with EdU fluorescent dye. Nuclei were counterstained with DAPI. Images were acquired using a confocal microscope, and the proportion of EdU‐positive cells was quantified.

### 2.10 Transwell Migration Assay

Cell migration was assessed using Transwell chambers (Corning, Corning, NY, USA). Cells (2 × 10^4^) were suspended in 100 μL serum‐free medium and seeded in the upper chamber. The lower chamber contained 600 μL complete medium with 10% FBS. After incubation for 24 h at 37°C, cells were fixed and stained with 0.1% crystal violet (Servicebio). Non‐migrated cells on the upper membrane surface were removed, and migrated cells on the lower surface were photographed using an IX73 inverted microscope (Olympus, Tokyo, Japan). Cells from five random fields were counted.

### 2.11 Ferroptosis Assays

Intracellular Fe^2+^ levels were measured using an Fe^2+^ assay kit (Elabscience, Wuhan, China). Cell lysates were centrifuged, and the supernatant was incubated with iron reductase reagent. Absorbance at 593 nm was measured, and Fe^2+^ concentration was calculated using a standard curve. Malondialdehyde (MDA) levels were measured using an MDA assay kit (Elabscience). Samples were incubated at 95°C for 40 min, then mixed with reaction solution. After centrifugation, absorbance at 532 nm was measured. For transmission electron microscopy (TEM), cells were fixed, sectioned, and stained with uranyl acetate. Mitochondrial ultrastructure was visualized using a transmission electron microscope (Hitachi High‐Technologies, Tokyo, Japan).

### 2.12 Fluorescence In Situ Hybridization (FISH)

A miR‐15a‐5p‐specific probe labeled with a red fluorescent dye was synthesized by Servicebio (Wuhan, China). FISH was performed using an RNA FISH Kit (GenePharma, Suzhou, China). Fresh tissue sections were fixed, digested with proteinase K, and dehydrated. After denaturation, probes were hybridized overnight. Sections were washed, and nuclei were counterstained with DAPI. Images were captured using a confocal microscope. FISH probe information is provided in Table S1.

### 2.13 Histology and Immunohistochemistry

Paraffin‐embedded brain tissues were sectioned, deparaffinized, and rehydrated. Antigen retrieval was performed in 10 mM sodium citrate buffer (pH 6.0). Hematoxylin and eosin (H&E) staining was performed using a commercial kit (Servicebio). For immunohistochemistry (IHC), sections were blocked with goat serum (Zhongshan Jinqiao, Beijing, China) and incubated with primary antibodies overnight, followed by secondary antibody incubation. Signals were visualized using a DAB substrate kit (Zhongshan Jinqiao), and nuclei were counterstained with hematoxylin (Cyagen Biosciences, Wuhan, China). Images were captured using an Olympus IX73 microscope. Five randomly selected fields per section were analyzed using ImageJ. Ki67 and GPX4 staining were quantified by counting positive cells, whereas GLS2 staining was quantified by measuring the positive staining area. Detailed antibody information is listed in Table S1.

### 2.14 In Vivo Xenograft Model

Four‐week‐old male BALB/c nude mice (*n* = 72) were obtained from GemPharmatech (Jiangsu, China). Under sevoflurane anesthesia, mice were stereotactically injected with 1 × 10^6^ LN229‐Luc cells into the right striatum (2 mm posterior to bregma, 3.5 mm lateral to the midline, and 3 mm depth). For miR‐15a‐5p knockdown experiments, mice were randomly divided into miR‐sh and miR‐shNC groups (*n* = 12 each). For co‐knockdown experiments, mice were assigned to 4 groups: miR‐shNC + GLS2‐shNC, miR‐sh + GLS2‐shNC, miR‐shNC + GLS2‐sh, and miR‐sh + GLS2‐sh (*n* = 12 per group). Tumor growth was monitored by bioluminescence imaging using an IVIS Spectrum (PerkinElmer, Waltham, MA, USA) on Days 7, 14, and 21 post‐injection. Mice with equivalent tumor signals on Day 7 were selected (*n* = 9 per group) and randomly allocated for Western blot, histology, or longitudinal imaging (*n* = 3 per group).

### 2.15 Statistical Analysis

Data are presented as mean ± SD from at least three independent experiments. Comparisons between two groups were performed using two‐tailed Student's *t*‐test. Multiple group comparisons were analyzed by one‐way or two‐way ANOVA with appropriate post hoc tests (Bonferroni for two‐way, Tukey for one‐way). Significance was defined as *p* < 0.05. Analyses were performed using GraphPad Prism 10 (GraphPad Software, Boston, MA, USA).

## 3 Results

### 3.1 miR‐15a‐5p Is Highly Expressed in GBM and Associated With Poor Prognosis

**FIGURE 1:**
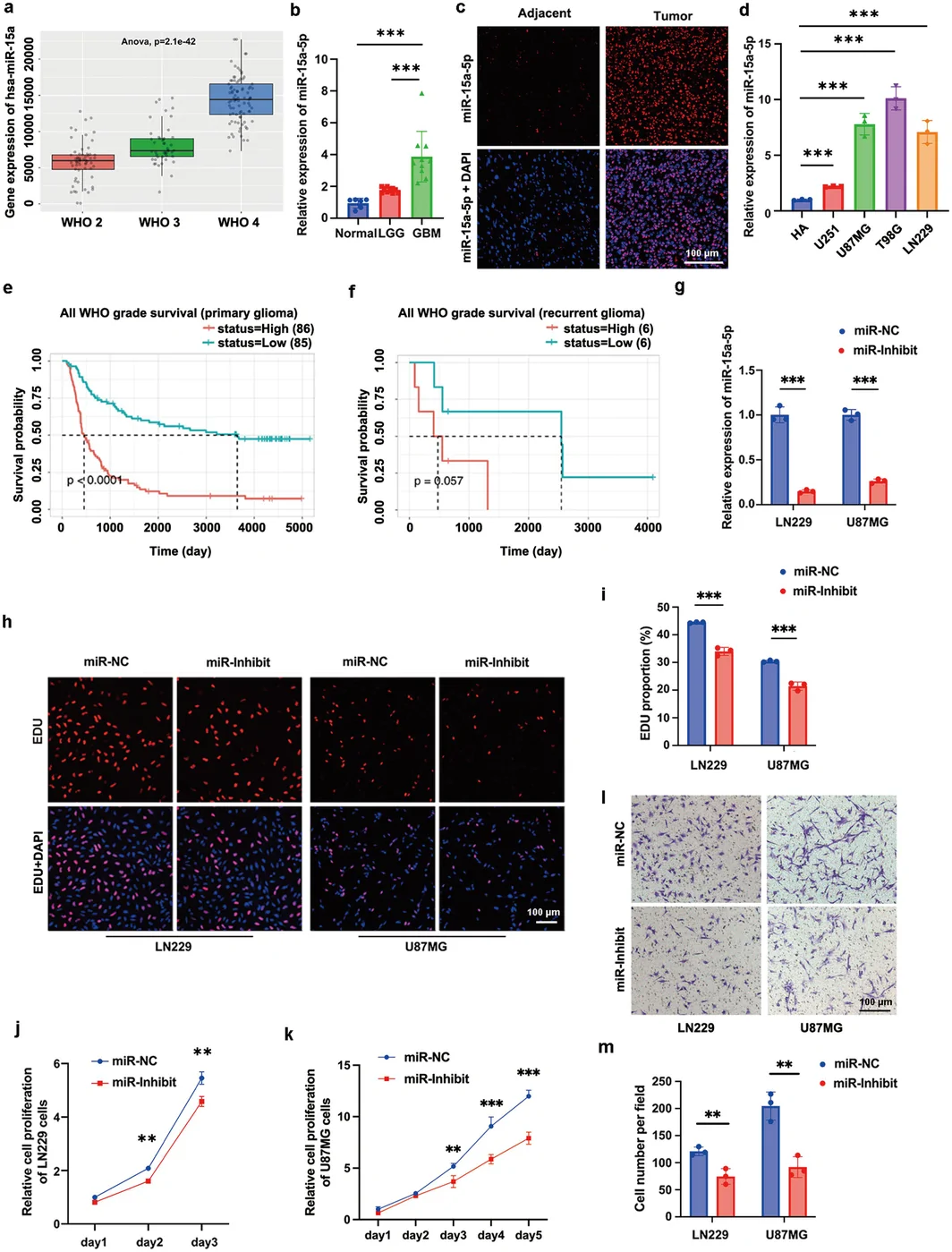
miR‐15a‐5p is upregulated in glioblastoma (GBM) and associated with poor prognosis. (a) Analysis of miR‐15a expression levels across different glioma grades based on the Chinese Glioma Genome Atlas (CGGA). (b) Expression levels of miR‐15a‐5p in non‐tumor tissues, low‐grade glioma (LGG), and glioblastoma (GBM) samples. (c) Representative FISH images showing miR‐15a‐5p expression in adjacent tissues and tumor tissues (scale bar = 100 μm). (d) miR‐15a‐5p expression levels in human astrocytes (HA) and GBM cell lines (U251, U87MG, T98G, and LN229). (e, f) Kaplan–Meier survival analysis of primary and recurrent glioma patients stratified by high and low miR‐15a expression (log‐rank test). (g) Knockdown efficiency of miR‐15a‐5p verified by RT‐qPCR in LN229 and U87MG cells. (h, i) Representative EdU staining images and quantitative analysis assessing cell proliferation following miR‐15a‐5p inhibition (scale bar = 100 μm). (j, k) CCK‐8 assay indicating LN229 and U87MG cell proliferation following miR‐15a‐5p inhibition. (l, m) Representative Transwell migration images and quantitative analysis evaluating migratory capacity after miR‐15a‐5p inhibition (scale bar = 100 μm). Data are presented as mean ± SD (*n* = 3). *p < 0.05, **p < 0.01, ***p < 0.001.

Analysis of the CGGA database revealed a positive association between miR‐15a expression and advanced WHO grade (Figure 1a). RT‐qPCR analysis of clinical samples confirmed that miR‐15a‐5p expression increased with tumor grade (Figure 1b). FISH analysis demonstrated stronger miR‐15a‐5p fluorescence signals in tumor tissues compared to adjacent non‐tumor tissues (Figure 1c). At the cellular level, miR‐15a‐5p expression was higher in all GBM cell lines (U251, U87MG, T98G, and LN229) than in the astrocytes (HA) (Figure 1d). Survival analysis indicated that patients with primary gliomas exhibiting higher miR‐15a levels had significantly shorter overall survival (Figure 1e). Patients with recurrent gliomas showed a similar trend, although the difference did not reach statistical significance (Figure 1f). Together, these data establish that miR‐15a‐5p is elevated in GBM and correlates with poor patient outcomes.

### 3.2 miR‐15a‐5p Promotes GBM Cell Proliferation and Migration

To investigate the functional role of miR‐15a‐5p in GBM, LN229, and U87MG cells were transfected with a miR‐15a‐5p inhibitor. RT‐qPCR confirmed effective knockdown (Figure 1g). EdU incorporation assays showed that miR‐15a‐5p inhibition significantly reduced the percentage of proliferating cells in both cell lines (Figure 1h,i). CCK‐8 assays further demonstrated that miR‐15a‐5p knockdown markedly suppressed cell proliferation over 72 h (Figure 1j,k). Transwell migration assays revealed that inhibition of miR‐15a‐5p significantly decreased the number of migrated cells in both cell lines (Figure 1l,m). These results indicate that miR‐15a‐5p promotes GBM cell proliferation and migration in vitro.

### 3.3 miR‐15a‐5p Knockdown Induces Ferroptosis in GBM Cells

**FIGURE 2:**
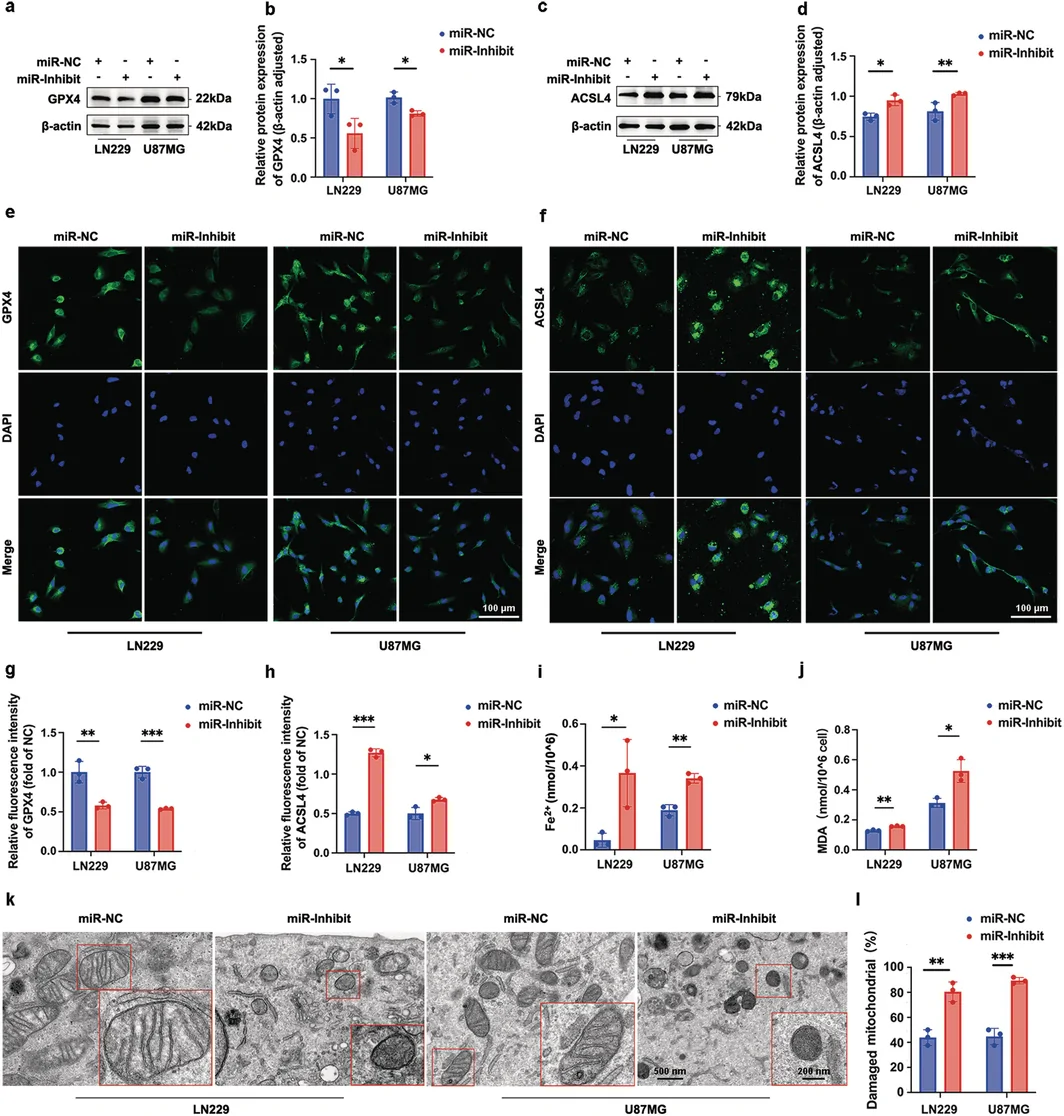
Inhibition of miR‐15a‐5p enhances ferroptosis in GBM cells. (a–d) Western blot (WB) analysis and quantitative measurement of GPX4 and ACSL4 expression in LN229 and U87MG cells. (e–h) Immunofluorescence (IF) images and quantitative analysis of GPX4 and ACSL4 expression (scale bar = 100 μm). (i) Intracellular Fe^2+^ levels measured by Fe^2+^ assay. (j) Lipid peroxidation assessed by MDA assay. (k, l) Transmission electron microscopy (TEM) images and quantitative analysis of mitochondrial ultrastructural alterations in LN229 and U87MG cells following miR‐15a‐5p inhibition. Representative images show ferroptosis‐associated features, including reduced mitochondrial size, increased membrane density, and loss of cristae (scale bar = 500 nm for low magnification and 200 nm for high magnification images). The proportion of damaged mitochondria is quantified in (l). Data are presented as mean ± SD (*n* = 3). *p < 0.05, **p < 0.01, ***p < 0.001 versus miR‐NC group.

To determine whether miR‐15a‐5p regulates cell death in GBM, a panel of markers representing major cell death pathways was first examined. Western blot analysis showed no significant changes in key markers of autophagy (P62), necroptosis (MLKL), pyroptosis (GSDMD), apoptosis (Bax), or cuproptosis (FDX1) following miR‐15a‐5p knockdown (Figure S3a–d), suggesting that miR‐15a‐5p does not broadly influence these forms of cell death. Given the emerging role of ferroptosis in GBM, the possibility that miR‐15a‐5p regulates this pathway was then investigated. In miR‐15a‐5p knockdown cells, Western blot analysis revealed significantly reduced GPX4 protein levels (Figure 2a,b) and increased ACSL4 expression (Figure 2c,d). Immunofluorescence confirmed decreased GPX4 fluorescence intensity (Figure 2e,g) and increased ACSL4 fluorescence intensity (Figure 2f,h) in the miR‐Inhibit group compared to controls. Biochemical assays further demonstrated that miR‐15a‐5p suppression significantly elevated intracellular Fe^2+^ levels (Figure 2i) and MDA content (Figure 2j), indicating enhanced lipid peroxidation. TEM revealed characteristic ferroptosis‐associated mitochondrial morphological alterations in the miR‐Inhibit group, including reduced mitochondrial volume, diminished cristae, and increased membrane density (Figure 2k,l). To verify the specificity of ferroptosis involvement, we treated miR‐15a‐5p‐inhibited GBM cells with Fer‐1. Fer‐1 treatment partially restored the suppressed proliferation and migration and reduced the elevated lipid peroxidation levels induced by miR‐15a‐5p inhibition in both LN229 and U87MG cells (Figure S4). Collectively, these findings indicate that miR‐15a‐5p knockdown specifically induces ferroptosis in GBM cells.

### 3.4 miR‐15a‐5p Directly Targets GLS2 and Negatively Regulates Its Expression

**FIGURE 3:**
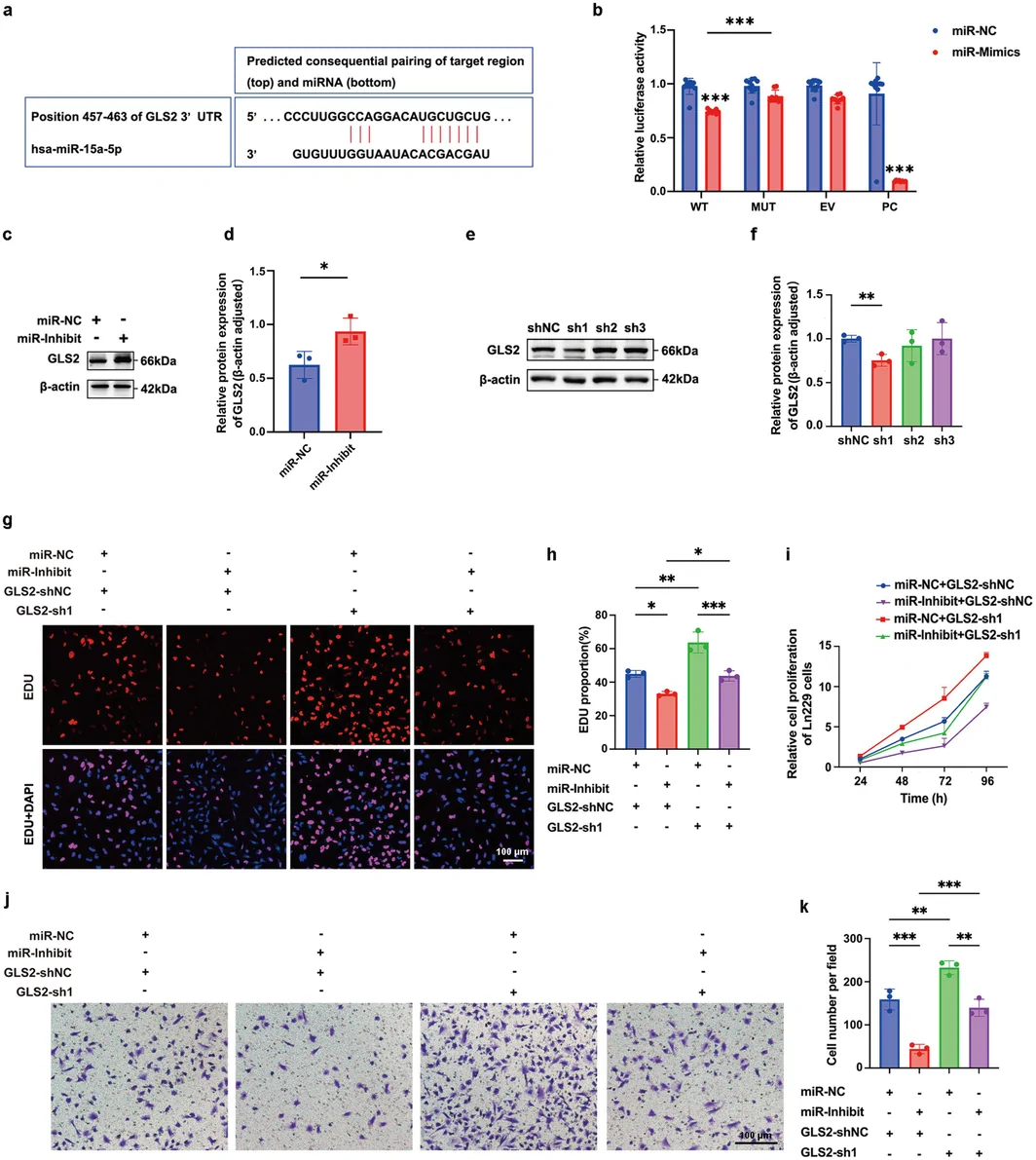
The miR‐15a‐5p/GLS2 axis regulates the malignant proliferation and migration phenotype of GBM. (a) Predicted binding site sequence between miR‐15a‐5p and the 3′UTR of GLS2. (b) Dual‐luciferase reporter assay showing relative luciferase activity of GLS2‐WT and GLS2‐MUT constructs following co‐transfection with miR‐15a‐5p mimics. (c, d) WB analysis and quantitative measurement of GLS2 expression in LN229 cells after miR‐15a‐5p inhibition. (e, f) WB analysis and quantitative measurement of GLS2 expression in LN229 cells following lentiviral‐mediated GLS2 knockdown. (g–k) Rescue experiments were performed in LN229 cells. (g, h) Representative EdU staining images and quantitative analysis assessing cell proliferation in rescue experiments (scale bar = 100 μm). (i) CCK‐8 assay demonstrating cell proliferation in the rescue experiment. (j, k) Representative Transwell migration images and quantitative analysis evaluating cell migration capacity in rescue experiments (scale bar = 100 μm). Data are presented as mean ± SD (*n* = 3). *p < 0.05, **p < 0.01, ***p < 0.001. Statistical comparisons were performed between the indicated groups.

TargetScan analysis predicted a conserved miR‐15a‐5p binding site in the GLS2 3′UTR (Figure 3a). IHC analysis showed that GLS2 expression was lower in GBM tissues compared to peritumoral tissues (Figure S1a). Western blot confirmed that GLS2 expression was significantly lower in GBM cell lines than in human astrocytes (Figure S1b,c). Dual‐luciferase reporter assays demonstrated that co‐transfection with miR‐15a‐5p mimics significantly decreased luciferase activity of the GLS2‐WT construct but had no effect on the GLS2‐MUT construct (Figure 3b), confirming direct binding of miR‐15a‐5p to the GLS2 3′UTR. Western blot analysis showed that GLS2 protein levels were significantly elevated following miR‐15a‐5p silencing (Figure 3c,d), indicating negative regulation of GLS2 by miR‐15a‐5p.

### 3.5 GLS2 Mediates miR‐15a‐5p–Induced Malignant Phenotypes and Ferroptosis Suppression

**FIGURE 4:**
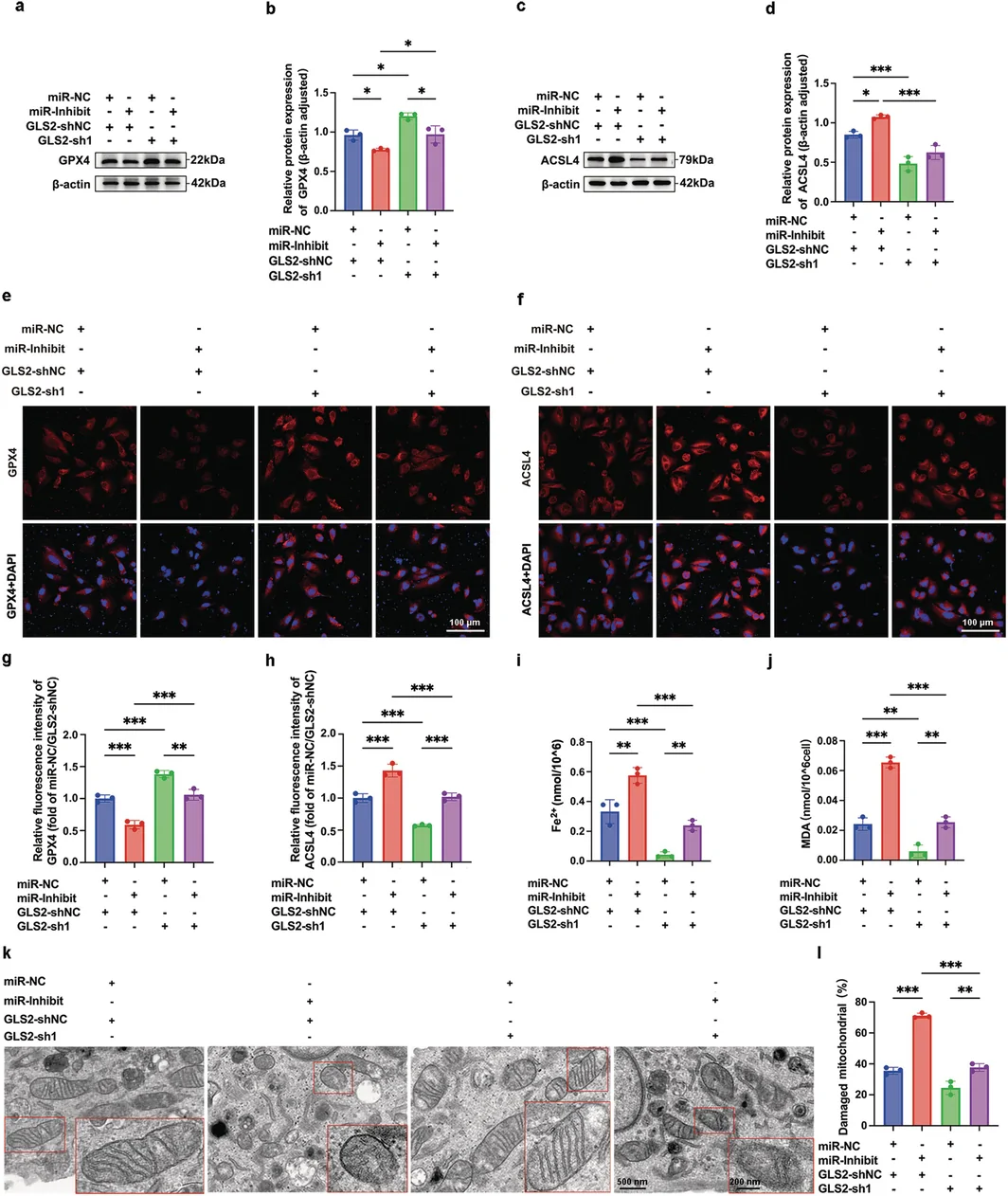
**miR‐15a‐5p regulates ferroptosis in GBM cells via the GLS2 axis. a‐d** WB analysis and quantitative measurement of GPX4 and ACSL4 expression in LN229 cells under rescue conditions. **e‐h** IF images and quantitative analysis of GPX4 and ACSL4 expression (scale bar = 100 μm). **i** Intracellular Fe^2+^ levels assessed by Fe^2+^ assay. **j** Lipid peroxidation measured by MDA assay. **k, l** TEM images and quantitative analysis of mitochondrial ultrastructural alterations under rescue conditions (scale bar = 500 nm for low magnification and 200 nm for high magnification images). Data are presented as mean ± SD (*n* = 3). *p < 0.05, **p < 0.01, ***p < 0.001. Statistical comparisons were performed between the indicated groups.

To validate the functional role of the miR‐15a‐5p/GLS2 axis, GLS2‐knockdown LN229 cells were established using lentiviral shRNA. Among three shRNAs tested, sh1 achieved the most efficient knockdown (Figure 3e,f) and was selected for subsequent rescue experiments in LN229 cells. In rescue assays, the inhibition of cell proliferation (EdU, CCK‐8) and migration (Transwell) induced by miR‐15a‐5p knockdown was reversed by concurrent GLS2 knockdown (Figure 3g–k), indicating that GLS2 acts as a downstream effector of miR‐15a‐5p in regulating GBM malignant phenotypes. To determine whether this axis functions through ferroptosis, ferroptosis markers were assessed. GLS2 knockdown reversed the enhanced ferroptosis phenotype induced by miR‐15a‐5p knockdown, as evidenced by restored GPX4 expression (Figure 4a,b,e,g), decreased ACSL4 expression (Figure 4c,d,f,h), reduced intracellular Fe^2+^ and MDA levels (Figure 4i,j), and restored mitochondrial morphology (Figure 4k,l). These findings demonstrate that GLS2 mediates miR‐15a‐5p–associated regulation of ferroptosis and malignant phenotypes in GBM cells.

### 3.6 miR‐15a‐5p Knockdown Induces Ferroptosis and Suppresses Tumor Growth In Vivo

**FIGURE 5:**
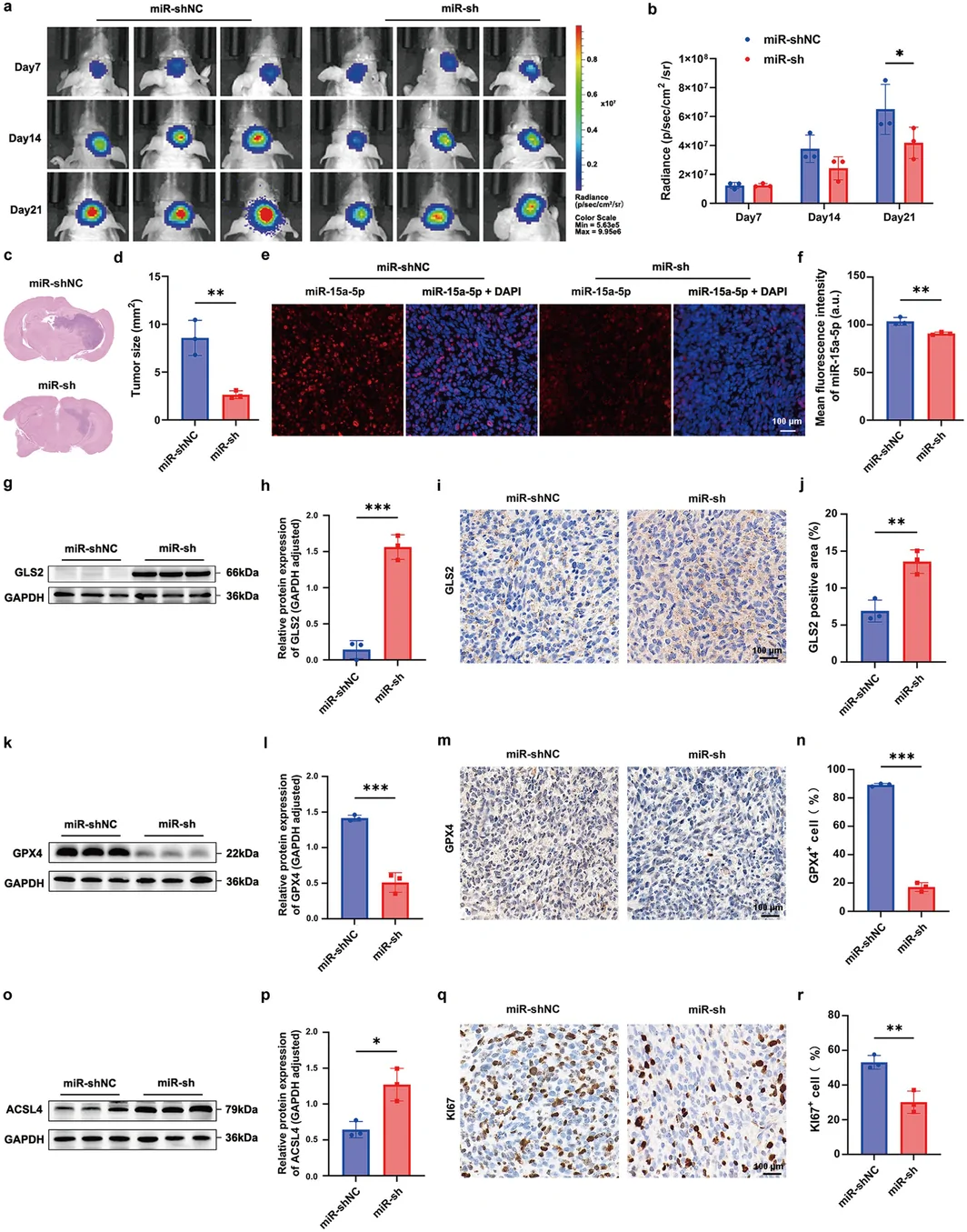
Tumor growth and ferroptosis assessment in intracranial xenograft models with stable miR‐15a‐5p knockdown. (a) Representative bioluminescence images of mice at Days 7, 14, and 21 post‐tumor implantations. (b) Quantitative analysis of tumor radiance (p/s/cm^2^/sr) at Days 7, 14, and 21. (c, d) Representative H&E‐stained images showing histological features of tumor tissues and quantitative analysis of tumor size. (e, f) Representative FISH images showing miR‐15a‐5p expression in tumor tissues from miR‐shNC and miR‐sh groups and corresponding quantification of mean fluorescence intensity (scale bar = 100 μm). (g, h) WB analysis and quantitative measurement of GLS2 expression in tumor tissues. (i, j) IHC images showing GLS2 expression in tumor tissues and quantitative analysis of GLS2 expression based on IHC. (k, l) WB analysis and quantitative measurement of GPX4 expression in tumor tissues. (m, n) IHC images showing GPX4 expression in tumor tissues and quantitative analysis of GPX4 expression based on IHC. (o, p) WB analysis and quantitative measurement of ACSL4 expression in tumor tissues. (q, r) IHC images showing Ki67 expression in tumor tissues and quantitative analysis of Ki67 expression based on IHC. (Scale bar of IHC = 100 μm). Data are presented as mean ± SD (*n* = 3 per group). *p < 0.05, **p < 0.01, ***p < 0.001 versus miR‐shNC group.

To assess the in vivo effects of miR‐15a‐5p knockdown, a stable miR‐15a‐5p knockdown LN229‐Luc cell line was established. Knockdown efficiency was confirmed by RT‐qPCR (Figure S2a). Intracranial xenograft models were established via stereotaxic injection. Bioluminescence imaging on Days 7, 14, and 21 post‐injection revealed significantly lower tumor signals in the miR‐sh group compared to the miR‐shNC group at Day 21 (Figure 5a,b). H&E staining showed smaller tumor volumes and sparser tissue architecture in the miR‐sh group (Figure 5c,d), and IHC analysis demonstrated significantly decreased Ki67 positivity (Figure 5q,r), confirming reduced tumor proliferation upon miR‐15a‐5p knockdown. FISH analysis confirmed effective knockdown of miR‐15a‐5p in tumor tissues from the miR‐sh group (Figure 5e,f). Western blot and IHC analyses revealed increased GLS2 protein expression in tumor tissues following miR‐15a‐5p knockdown (Figure 5g–j). Consistent with in vitro findings, miR‐15a‐5p knockdown induced ferroptosis in vivo, as indicated by decreased GPX4 expression (Figure 5k,l) and increased ACSL4 expression (Figure 5o,p) by Western blot, and confirmed by IHC showing significantly lower GPX4 staining intensity (Figure 5m,n). Key markers of other cell death pathways showed no significant changes (Figure S3e,f), further supporting ferroptosis specificity.

### 3.7 GLS2 Mediates miR‐15a‐5p–Regulated Tumor Growth and Ferroptosis In Vivo

**FIGURE 6:**
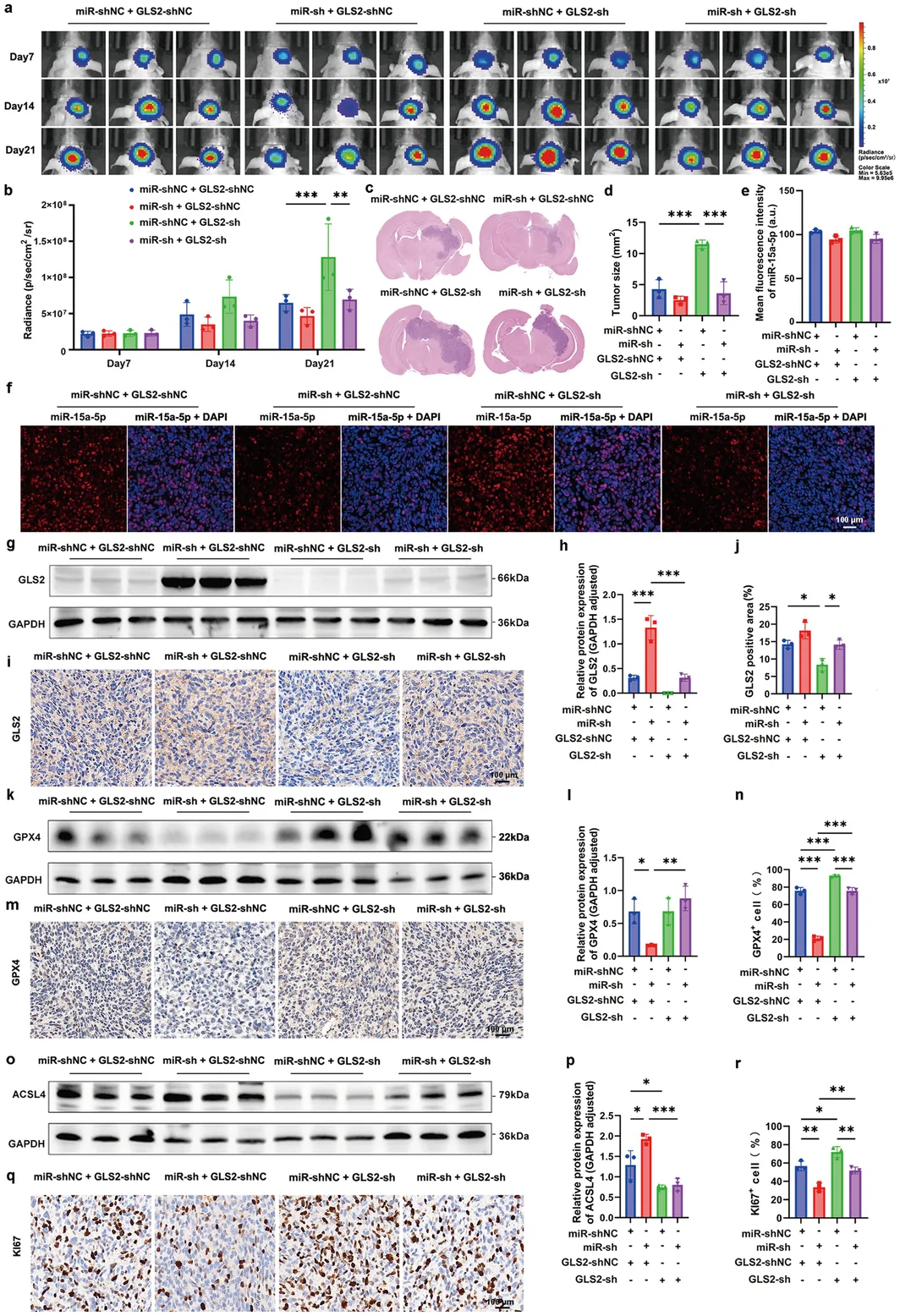
The miR‐15a‐5p/GLS2 axis regulates tumor growth and ferroptosis in intracranial xenograft models. (a) Representative bioluminescence images of mice at Days 7, 14, and 21 post‐tumor implantations. (b) Quantitative analysis of tumor radiance (p/s/cm^2^/sr) at Days 7, 14, and 21. (c, d) Representative H&E‐stained images showing histological features of tumor tissues and quantitative analysis of tumor size. (e, f) Representative FISH images showing miR‐15a‐5p expression in tumor tissues from rescue experiment groups and corresponding quantification of mean fluorescence intensity (scale bar = 100 μm). (g，h) WB analysis and quantitative measurement of GLS2 expression in tumor tissues. (i, j) IHC images showing GLS2 expression in tumor tissues and quantitative analysis of GLS2 expression based on IHC. (k, l) WB analysis and quantitative measurement of GPX4 expression in tumor tissues. (m, n) IHC images showing GPX4 expression in tumor tissues and quantitative analysis of GPX4 expression based on IHC. (o, p) WB analysis and quantitative measurement of ACSL4 expression in tumor tissues. (q, r) IHC images showing Ki67 expression in tumor tissues and quantitative analysis of Ki67 expression based on IHC. (Scale bar of IHC = 100 μm). Data are presented as mean ± SD (*n* = 3 per group). *p < 0.05, **p < 0.01, ***p < 0.001. Statistical comparisons were performed between the indicated groups.

To determine whether GLS2 mediates the in vivo effects of miR‐15a‐5p, rescue experiments were performed using stable cell lines with co‐knockdown of miR‐15a‐5p and GLS2. RT‐qPCR and Western blot confirmed that miR‐15a‐5p knockdown upregulated GLS2 expression, and simultaneous GLS2 knockdown reversed this upregulation (Figure S2c,d). GLS2 knockdown did not significantly affect miR‐15a‐5p expression (Figure S2b), supporting a downstream regulatory relationship. In vivo imaging showed that concurrent GLS2 knockdown significantly reversed the tumor growth inhibition induced by miR‐15a‐5p knockdown at Day 21 post‐injection (Figure 6a,b). H&E staining and Ki67 IHC demonstrated that GLS2 co‐knockdown reversed miR‐15a‐5p knockdown–induced phenotypes, including reduced tumor burden and decreased proliferative activity (Figure 6c,d,q,r). FISH and Western blot/IHC confirmed successful in vivo modulation of miR‐15a‐5p and GLS2 (Figure 6e–j). At the ferroptosis level, concurrent GLS2 knockdown reversed miR‐15a‐5p–induced alterations in ferroptosis‐related proteins, as indicated by increased GPX4 expression (Figure 6k–n) and decreased ACSL4 expression (Figure 6o,p). No significant alterations were observed in markers of other cell death pathways (Figure S3g,h). These in vivo rescue data confirm that GLS2 mediates miR‐15a‐5p–associated regulation of ferroptosis and tumor growth in GBM.

## 4 Discussion

The current study shows that miR‐15a‐5p regulates ferroptosis in GBM and its overexpression promotes tumor progression by directly suppressing GLS2. These findings extend the current understanding of miR‐15a‐5p by linking it to ferroptosis regulation in GBM, a process that has not been previously explored in this context. Notably, this study emphasizes the context‐dependent nature of miRNA function in ferroptosis regulation, implying that the impact of a single miRNA may vary significantly depending on tumor type and cellular environment. In this setting, the miR‐15a‐5p/GLS2 axis represents a previously unknown regulatory mechanism that controls ferroptosis in GBM (The Graphical Abstract provides an overview of the suggested regulatory mechanism).

MicroRNAs are increasingly recognized as highly versatile regulators whose functions are dictated by cellular context rather than being fixed properties [21, 22, 23]. A growing body of evidence indicates that miRNA–target interactions are highly dynamic and can produce distinct, even opposing, biological outcomes depending on the availability of target transcripts and the cellular environment [24]. The findings of the present study further support this concept. While miR‐15a‐5p has been reported to promote ferroptosis in non‐neoplastic contexts through targeting GPX4 [18, 19], we demonstrate that in GBM it suppresses ferroptosis via a distinct mechanism involving GLS2. This functional divergence underscores that miRNA‐mediated regulation of ferroptosis is highly context‐dependent and highlights the necessity of investigating these regulatory relationships within specific tumor settings. In glioma, previous studies have linked miR‐15a‐5p to proliferation and invasion [20], but without addressing ferroptosis. By integrating these observations, our study expands the functional scope of miR‐15a‐5p in GBM by identifying ferroptosis regulation as an additional layer of its oncogenic activity.

Glutaminase 2 (GLS2) is a mitochondrial enzyme that catalyzes the conversion of glutamine to glutamate, a central step in glutamine metabolism [25, 26]. The downstream metabolic consequences of GLS2 activity can influence ferroptosis in complex and context‐dependent ways, as glutamate supports glutathione biosynthesis while also contributing to reactive oxygen species generation through mitochondrial metabolism [16, 27, 28]. In GBM, where ferroptosis resistance is commonly observed and anti‐ferroptotic pathways such as GPX4 and SLC7A11 are frequently upregulated [29, 30], suppression of GLS2 may represent an additional regulatory layer limiting ferroptosis [31, 32]. Importantly, GLS2 downregulation has been reported to occur independently of p53 status in GBM [33], suggesting that multiple regulatory mechanisms converge on this molecule. The present study identifies miR‐15a‐5p as one such post‐transcriptional regulator. Collectively, these findings suggest that GLS2 should not be viewed merely as a downstream target, but rather as a functionally important node linking glutamine metabolism and ferroptosis regulation in GBM.

Ferroptosis specificity was verified by the lack of alterations in other cell death pathway indicators, and the functional importance of the miR‐15a‐5p/GLS2 axis was thoroughly validated utilizing complementary loss‐of‐function and rescue approaches in vitro and in vivo. These results confirm the miR‐15a‐5p/GLS2 axis as a true regulator of ferroptosis in GBM.

### 4.1 Limitations of This Study

First, the upstream mechanisms driving miR‐15a‐5p overexpression in GBM remain unclear. Second, although key markers of other cell death pathways showed no significant changes, the possibility that miR‐15a‐5p may regulate other forms of cell death under different conditions cannot be completely excluded. Third, miR‐15a‐5p may also influence other cancer‐relevant processes—such as metabolism, stemness, or immune evasion—through additional downstream targets beyond GLS2. Fourth, although our findings suggest a clinical association between miR‐15a‐5p expression and glioma progression, particularly in GBM, further validation in larger patient cohorts with comprehensive clinical annotations and grade‐stratified analyses is required to determine its clinical significance. Despite these limitations, they do not affect the central conclusion that the miR‐15a‐5p/GLS2 axis regulates ferroptosis in GBM.

## 5 Conclusions

In summary, this study demonstrates that miR‐15a‐5p promotes GBM progression by directly suppressing GLS2, thereby inhibiting ferroptosis. These findings define a context‐dependent regulatory axis and provide new insight into the role of miRNA‐mediated ferroptosis regulation in GBM. Given the urgent need for effective therapies, targeting the miR‐15a‐5p/GLS2 axis may represent a potential strategy for ferroptosis‐based intervention.

## Funding

This work was supported by Jiangxi Provincial Health Technology Project; Science and Technology Project of Jiangxi Provincial Administration of Traditional Chinese Medicine (2024B1160); Ganzhou Science and Technology Plan Project (GZ2024YLJ005 and 2023LNS17441).

## Disclosure

Sex as a biological variable: Both male and female patients were included in the clinical samples. However, due to the limited sample size and the primary focus of this study on mechanistic investigation, formal sex‐based subgroup analyses were not performed. In vivo experiments were conducted using male mice as part of a controlled experimental design for initial mechanistic evaluation. In vitro experiments were performed using established cell lines without incorporating sex as an experimental variable. Future studies with larger sample sizes and inclusion of both sexes in experimental models are warranted to further investigate potential sex‐specific differences.

## Ethics Statement

All procedures involving human participants were conducted in accordance with the Declaration of Helsinki and approved by the Ethics Committee of Ganzhou People's Hospital (Approval No. PJB2025‐376‐01). All animal experiments were approved by the Ethics Committee of Ganzhou People's Hospital (Approval No. PID2025‐005‐01) and conducted in compliance with national laboratory animal guidelines (Laboratory Animal Use Permit No.: SYXK (Gan) 2023‐0006). The maximal tumor size permitted by the institutional ethics committee was 1.5 cm in maximum diameter; no animals exceeded this limit.

## Consent

This study strictly adheres to regulations concerning the privacy rights of human subjects. All human subjects participating in the experiment have signed informed consent forms.

## Conflicts of Interest

The authors declare no conflicts of interest.

## Supporting information


**Figure S1:** Expression of GLS2 in glioblastoma (GBM). (a) Representative immunohistochemistry (IHC) images showing GLS2 expression in adjacent (peritumoral) and tumor tissues from a GBM patient (scale bar = 50 μm). (b, c) Western blot (WB) analysis and quantitative measurement of GLS2 expression in human astrocytes (HA) and glioblastoma (GBM) cell lines (U251, U87MG, T98G, and LN229). Data are presented as mean ± SD (*n* = 3). ***p < 0.001 versus HA group.
**Figure S2:** Validation of lentiviral transfection efficiency in LN229‐Luc cells. (a) RT ‐qPCR analysis showing the knockdown efficiency of miR‐15a‐5p in LN229‐Luc cells. (b) RT‐qPCR analysis confirming miR‐15a‐5p expression levels in the rescue experiment groups. (c, d) WB analysis and quantitative measurement of GLS2 expression in LN229‐Luc cells under rescue conditions. Data are presented as mean ± SD (*n* = 3). *p < 0.05, **p < 0.01, ***p < 0.001. Statistical comparisons were performed between the indicated groups.
**Figure S3:** Detection of canonical protein markers associated with different modes of cell death. (a, b) Representative WB images and quantitative analysis of the indicated cell death–related proteins in LN229 cells following miR‐15a‐5p inhibition. (c, d) Representative WB images and quantitative analysis of the indicated cell death–related proteins in U87MG cells following miR‐15a‐5p inhibition. (e, f) Representative WB images and quantitative analysis of the indicated proteins in mouse tumor tissues after stable knockdown of miR‐15a‐5p. (g, h) Representative WB images and quantitative analysis of the indicated proteins in mouse tumor tissues from the rescue experiment. Data are presented as mean ± SD (*n* = 3). No significant differences were observed between the indicated groups.
**Figure S4:** Ferrostatin‐1 rescues miR‐15a‐5p inhibition‐induced malignant phenotypes and lipid peroxidation in GBM cells. (a, b) Representative EdU staining images and quantitative analysis assessing LN229 cell proliferation after miR‐15a‐5p inhibition with or without Ferrostatin‐1 (Fer‐1) treatment (scale bar = 100 μm). (c) CCK‐8 assay showing LN229 cell proliferation under the same conditions. (d, e) Representative EdU staining images and quantitative analysis assessing U87MG cell proliferation after Fer‐1 treatment (scale bar = 100 μm). (f) CCK‐8 assay showing U87MG cell proliferation under the indicated treatment conditions. (g, h) Representative Transwell migration images and quantitative analysis evaluating LN229 cell migration capacity after Fer‐1 rescue (scale bar = 100 μm). (i) Lipid peroxidation levels measured by MDA assay in LN229 cells. (j, k) Representative Transwell migration images and quantitative analysis evaluating U87MG cell migration capacity after Fer‐1 treatment (scale bar = 100 μm). (l) Lipid peroxidation levels measured by MDA assay in U87MG cells. Data are presented as mean ± SD (*n* = 3). *p < 0.05, **p < 0.01, ***p < 0.001. Statistical comparisons were performed between the indicated groups.

**Table S1:** List of primers, antibodies, lentiviruses, and probes.

## Data Availability

All data generated or analyzed during this study are included in this published article and its supplement information files. Additional data are available from the corresponding authors upon reasonable request.
