# Supplementary material for: miR‐15a‐5p Regulates Ferroptosis in Glioblastoma by Targeting GLS2
Source: CNS Neurosci Ther. 2026 Aug 30;32(9):e71097. doi: 10.1002/cns.71097 (PMC13526403; doi:10.1002/cns.71097)
Supplement: Supplementary file 1 [file CNS-32-e71097-s002.docx]

**Supplementary Fig. S1** **Expression of GLS2 in glioblastoma (GBM).** **a** Representative immunohistochemistry (IHC) images showing GLS2 expression in adjacent (peritumoral) and tumor tissues from a GBM patient (scale bar = 50 μm). **b, c** Western blot (WB) analysis and quantitative measurement of GLS2 expression in human astrocytes (HA) and glioblastoma (GBM) cell lines (U251, U87MG, T98G, LN229). Data are presented as mean ± SD (n = 3). ***P < 0.001 vs. HA group.

**Supplementary Fig. S2 Validation of lentiviral transfection efficiency in LN229-Luc cells.** **a** RT -qPCR analysis showing the knockdown efficiency of miR-15a-5p in LN229-Luc cells. **b** RT-qPCR analysis confirming miR-15a-5p expression levels in the rescue experiment groups. **c, d** WB analysis and quantitative measurement of GLS2 expression in LN229-Luc cells under rescue conditions. Data are presented as mean ± SD (n = 3). *P < 0.05, **P < 0.01, ***P < 0.001. Statistical comparisons were performed between the indicated groups.

**Supplementary Fig. S3 Detection of canonical protein markers associated with different modes of cell death. a,b** Representative WB images and quantitative analysis of the indicated cell death–related proteins in LN229 cells following miR-15a-5p inhibition. **c,d** Representative WB images and quantitative analysis of the indicated cell death–related proteins in U87MG cells following miR-15a-5p inhibition. **e,f** Representative WB images and quantitative analysis of the indicated proteins in mouse tumor tissues after stable knockdown of miR-15a-5p. **g,h** Representative WB images and quantitative analysis of the indicated proteins in mouse tumor tissues from the rescue experiment. Data are presented as mean ± SD (n = 3). No significant differences were observed between the indicated groups

**Supplementary Fig. S4 Ferrostatin-1 rescues miR-15a-5p inhibition-induced malignant phenotypes and lipid peroxidation in GBM cells. a, b** Representative EdU staining images and quantitative analysis assessing LN229 cell proliferation after miR-15a-5p inhibition with or without Ferrostatin-1 (Fer-1) treatment (scale bar = 100 μm). **c** CCK-8 assay showing LN229 cell proliferation under the same conditions. **d, e** Representative EdU staining images and quantitative analysis assessing U87MG cell proliferation after Fer-1 treatment (scale bar = 100 μm). **f** CCK-8 assay showing U87MG cell proliferation under the indicated treatment conditions. **g, h** Representative Transwell migration images and quantitative analysis evaluating LN229 cell migration capacity after Fer-1 rescue (scale bar = 100 μm). **i** Lipid peroxidation levels measured by MDA assay in LN229 cells. **j, k** Representative Transwell migration images and quantitative analysis evaluating U87MG cell migration capacity after Fer-1 treatment (scale bar = 100 μm). **l** Lipid peroxidation levels measured by MDA assay in U87MG cells. Data are presented as mean ± SD (n = 3). *P < 0.05, **P < 0.01, ***P < 0.001. Statistical comparisons were performed between the indicated groups.
